# Targeting Underglycosylated MUC1 for the Selective Capture of Highly Metastatic Breast Cancer Cells Under Flow

**DOI:** 10.1007/s12195-013-0282-y

**Published:** 2013-05-22

**Authors:** Yue Geng, Tait Takatani, Kimberly Yeh, Jong-Wei Hsu, Michael R. King

**Affiliations:** Department of Biomedical Engineering, Cornell University, 205 Weill Hall, 526 Campus Rd, Ithaca, NY 14853 USA

**Keywords:** E-selectin, P-selectin, L-selectin, MUC1, ICAM-1, Glycosylation, Sialyl Lewis x, Breast cancer, Circulating tumor cells

## Abstract

The underglycosylated form of the MUC1 glycoprotein, uMUC1, has been identified as a ligand for both E-selectin and ICAM-1 and can play multiple potential roles during rolling and firm adhesion events in the metastatic cascade. Using flow cytometry and confocal microscopy, the T47D and ZR-75-1 cell lines were verified to highly express uMUC1, however it was found that only ZR-75-1 cells expressed the E-selectin binding moiety sialyl Lewis x (sLe^x^). Furthermore, perfusing T47D cells through E-selectin coated microtubes resulted in fast rolling velocities and low numbers of interacting cells and blocking uMUC1 with the SM3 antibody had no effect. ZR-75-1 cells, on the other hand, were highly dependent on the E-selectin:uMUC1 interaction as exemplified by significant increases in cell rolling velocities and decreases in the number of interacting cells when blocking with SM3 or when uMUC1 expression was knocked down *via* siRNA transfection. Whereas uMUC1 interactions with E-selectin supported cell rolling, P-selectin: uMUC1 interactions exclusively facilitated cell tethering, while L-selectin surfaces supported no cell adhesive interactions. These experimental observations are consistent with molecular dynamics simulations of uMUC1 bound to E-, P-, and L-selectin where the degree of residue contact correlated with the differential adhesion of uMUC1 to each selectin. Finally, an E-selectin and SM3 combined surface coating captured approximately 30% of the total number of interacting cancer cells comparable to the number of adhered cells when utilizing E-selectin and ICAM-1 combined surfaces. The E-selectin/SM3 surface strategy offers a viable method to selectively capture cancer cells from whole blood samples.

## Introduction

Leukocyte recruitment to the inflamed endothelium and cancer metastasis through the bloodstream *via* circulating tumor cells (CTCs) have been proposed to share a similar stepwise mechanism that allows for cell adhesion and extravasation.^12^,^28^,^31^,^33^,^36^ Referred to as adhesion cascades (leukocyte and CTC), cells first tether and roll on the blood vessel wall *via* transient interactions between P- and E-selectin present on the inflamed endothelium^4^,^36^ and carbohydrate moieties, such sialyl Lewis x (sLe^x^) or sialyl Lewis a (sLe^a^) found on leukocytes and CTCs.^34^,^35^ Sufficiently slow cell rolling permits firm cell adhesion events mediated by endothelial intercellular adhesion molecule-1 (ICAM-1) at locations of transendothelial migration.^2^,^11^ Work by our group has capitalized on these selectin:carbohydrate based interactions to capture CTCs as well as hematopoietic stem and progenitor cells with the ability to maintain cell viability.^18^,^19^,^29^,^30^ Further differentiation between CTCs and contaminating leukocytes will allow isolation processes to be further optimized with respect to both yield and purity.

Three cell adhesion molecules constitute the members of the selectin family. E-selectin, primarily expressed by inflamed endothelial cells, has been extensively studied for its role in leukocyte recruitment in response to vascular injury^24^ as well as CTC adhesion.^12^,^20^ P-selectin is a granule protein expressed by both platelets and endothelial cells, and therefore has been linked to the adhesion of platelets,^15^ leukocytes,^25^ and cancer cells^21^ to the endothelium. L-selectin differs in that it is expressed by leukocytes, not endothelial cells, and therefore is not normally considered in the context of cancer cell adhesion. All selectins contain the epidermal growth factor and lectin domains where the carbohydrate moieties can bind *via* calcium dependent interactions.^26^ These carbohydrate moieties are attached to O-glycosylated proteins on the cell surface, referred to as selectin ligands, and in the context of cell adhesion to the vascular wall, both the metastatic and leukocyte adhesion cascades rely on similar selectin ligands to facilitate initial tethering and rolling events.^3^,^41^

Leukocytes express three main selectin ligands: P-selectin glycoprotein ligand 1 (PSGL-1), E-selectin ligand (ESL-1), and CD44.^17^ CTCs, on the other hand, not only potentially express these three selectin ligands^7^,^39^ but also a myriad of other selectin ligands such as CD24, CD43, carcinoembryonic antigen (CEA), and podocalyxin-like protein (PCLP).^1^,^38^,^39^,^43^ Recently two novel E-selectin ligands, mainly present on breast cancer cells, have been postulated: Mac-2BP^32^ and MUC1.^10^,^42^,^43^ Interestingly, the underglycosylated form of MUC1 (uMUC1) has been shown to be highly expressed in various breast cancer cells^6^,^27^ and clinically, high uMUC1 expression is correlated to poor prognosis and increased metastases.^40^ Moreover, the core of MUC1 has also been shown to be an ICAM-1 ligand.^16^ Motivated by these findings, we recently elucidated the synergistic role of uMUC1 as both an E-selectin and ICAM-1 ligand during the CTC adhesion cascade.^14^

Although selectin ligands can potentially bind to all three selectins *via* calcium:carbohydrate dependent binding, selectin ligands often preferentially bind to particular selectins. Hidalgo *et al.* 17 clarified the specific functions of each selectin ligand during the leukocyte adhesion cascade. PSGL-1, which preferentially binds to P- and L-selectin,^35^ was found responsible for the initial capture and tethering of leukocytes. ESL-1, which preferentially binds to E-selectin,^37^ generated rolling cells from tethering events. Lastly, CD44 interactions with E-selectin have been found to control cell rolling velocity.^17^ L-selectin (present on leukocytes) is polarized after leukocyte firm adhesion where further possible interactions with PSGL-1 on other leukocytes in the bloodstream can occur. It is also possible for L-selectin to interact with E-selectin on the endothelium to further enhance cell capture.^44^ Our group has exploited this selectin preference while studying the efficiency of halloysite nanotubes on CTC capture,^18^,^19^ where P-selectin was utilized when cells expressed PSGL-1 and E-selectin was used otherwise. We have also found that pathologically decreasing extracellular pH can enhance PSGL-1 binding to P- and L-selectin, while having no effect on E-selectin:PSGL-1 binding.^5^ Taken together, the use of different selectins can offer another axis of control to more efficiently and selectively capture CTCs.

In our previous study of MUC1, we examined the adhesion profiles of ZR-75-1 and MCF7 cells on combined surfaces of E-selectin and ICAM-1.^14^ We found that whereas both cell lines exhibited high levels of MUC1, only ZR-75-1 cells exhibited uMUC1 which leads to slower rolling velocities (facilitated by E-selectin) and the existence of firm adhesion events (facilitated by ICAM-1) which strongly suggested that the uMUC1 glycoprotein significantly participates in the adhesion of metastatic cancer cells to the inflamed endothelium. To extend the E-selectin/ICAM-1 combined surface study in the context of cell adhesion facilitated by uMUC1, we first utilize the ZR-75-1 and T47D cell lines, which both have been shown to express high levels of uMUC1, to further characterize the differential adhesion of uMUC1 to E-selectin coated surfaces. We then explore the preferential binding of uMUC1 to E-, P-, and L-selectin *via* experimental rolling assays under shear stress and molecular dynamics (MD) simulations. Since uMUC1 is only expressed by CTCs in the bloodstream, we further hypothesize that utilizing a combined E-selectin and SM3 (antibody that specifically binds to uMUC1) surface may provide a novel approach to target CTCs for capture or treatment, where the E-selectin:uMUC1 interactions facilitate cell rolling and the SM3:uMUC1 interactions selectively capture rolling CTCs.

## Materials and Methods

### Reagents

Recombinant human E-selectin-IgG chimera was purchased from R&D systems (Minneapolis, MN). Blotting grade blocker non-fat dry milk was purchased from Bio-Rad Laboratories (Hercules, CA) and Protein-G was purchased from EMD Biosciences (San Diego, CA). FITC mouse anti-human CD227 (clone HPMV), FITC Mouse IgG1 k isotype control, purified mouse anti-human CD15s (clone CSLEX), APC rat anti-mouse IgM, FITC mouse anti-human CD44, and FITC goat anti-mouse IgG/IgM were all purchased from BD Biosciences (San Jose, CA). Mouse anti-human MUC1 mAb clone SM3 (recognizing the underglycosylated form of MUC1) was purchased from Abcam (Cambridge, MA). Mouse anti-human CA19-9 (sLe^a^) was purchased from Santa Cruz Biotechnology (Santa Cruz, CA). APC anti-human IgG antibodies were purchased from Invitrogen (Carmarillo, CA). Ca^2+^ and Mg^2+^ free DPBS (Invitrogen, Camarillo, CA, USA), calcium carbonate (Sigma Chemical Co., St. Louis, MO, USA), and low endotoxin (1 ng/mg), essentially globulin-free Bovine Serum Albumin (Sigma Chemical Co., St. Louis, MO, USA) were used to prepare flow buffer for cell adhesion assays. CELLview™ glass bottom dishes from Greiner Bio-One were used to plate cells for confocal microscopy experiments.

### Breast Cancer Cell Culture

Breast cancer cell lines ZR-75-1 and T47D were purchased from ATCC and maintained in 10% Fetal Bovine Serum (FBS; Cellgro), 1% penicillin–streptomycin (Invitrogen), and RPMI 1640 medium at 37 °C with 5% CO_2_ in a humidified incubator.

### Flow Cytometry

Cells were removed from tissue culture flasks prior to antibody incubation using an enzyme-free cell dissociation buffer solution. After washing with 1× DPBS, the cells were resuspended in 1% BSA in DPBS to a final concentration of approximately 250,000 cells per sample. Antibodies against MUC1 or appropriate isotype controls were added to the cell suspensions and incubated over ice for 45 min. Specifically, mouse anti-human MUC1 mAb clone HPMV (reacts with the peptide core of MUC1) and mouse anti-human MUC1 mAb clone SM3 (recognizing the underglycosylated form of MUC1) were used in this study. Antibodies against CD44, sLe^a^, and sLe^x^ were also used in this study to quantify the ligand and glycan expression on both cell types. Following incubation, cells were washed twice with 500 *μ*L of 1% BSA to remove any unbound antibody. Flow cytometry samples were analyzed using a BD Accuri C6 flow cytometer (Ann Arbor, MI).

### Confocal Immunofluorescence Microscopy

ZR-75-1 and T47D cells were plated in CELLview™ glass bottom dishes overnight prior to imaging. Surface staining for MUC1 and uMUC1 was performed using mouse anti-human CD227 mAb, mouse anti-human MUC1 mAb (clone SM3), and Alexa 647 rat anti-mouse mAb as a secondary antibody as described in Geng *et al.* 14 Cells were also incubated with conjugated human recombinant E-selectin chimera for 30 min at room temperature (RT). A Zeiss 710 laser scanning confocal microscope at the Cornell University microscopy and imaging core facility was used to collect images using a 40× objective.

### Preparation of Immobilized Protein Surfaces

Polyurethane microtubes with an inner diameter of 300 *μ*m (Braintree Scientific Inc., Braintree, MA, USA) were cut to a length of 50 cm. Recombinant human E-selectin-IgG chimeric protein was dissolved in 1× PBS to a final concentration of 5 *μ*g/mL. The microtube surface was first rinsed with 75% ethanol and then 1× PBS. The surface was subsequently incubated with 10 *μ*g/mL protein-G solution for 1.5 h, followed by a 2 h incubation with selectin chimera then blocked with 5% milk protein in PBS for 1 h. Control tubes were blocked with 5% milk protein in PBS for 1 h.

### Flow-Based Adhesion Assay

Microtubes functionalized with E-selectin/Fc chimera (described above) were taped onto a piece of thin glass in the viewing area of the Olympus IX81 inverted microscope (Olympus America Inc., Melville, NY, USA). A CCD camera (model no: KP-M1AN, Hitachi, Tokyo, Japan) and a DVD recorder (model no: DVD-1000MD, Sony Electronics) were used to record experiments for offline analysis. ZR-75-1 and T47D breast cancer cells suspended in flow buffer containing calcium were perfused through the microtubes using a syringe pump (KDS 230, IITC Life Science, Woodland Hills, CA) at a wall shear stress of 1.0 dyne/cm^2^.

### Neuraminidase Treatment

For certain experiments, ZR-75-1 and T47D cells were treated with 0.1 U/mL Vibrio Cholerae Neuraminidase (Roche Applied Science, Indianapolis, IN) for 45 min at 37 °C to cleave the terminal sialic acid residues. After enzyme treatment cells were washed and perfused through the functionalized microtubes.

### siRNA Transfection

Cells were transfected with 20 nM of MUC1 siRNA (QIAGEN) for 12 h. Total RNA was isolated from transfected cells using TRIzol (Life Technologies). Real-time PCR was carried out using oligo dT first strand primers, MUC1 specific primers [(5′-TGCATCAGGCTCAGCTTCTA-3′ and 5′-GAAATGGCACATCACTCACG-3′, IDT Integrated DNA Technologies], and SYBR Green PCR Kit (QIAGEN). MUC1 expression was normalized against GAPDH.

### Polymorphonuclear Neutrophil (PMN) and Buffy Coat Isolation

Human peripheral blood was collected from healthy adult donors after informed consent by venipuncture into BD Vacutainer tubes, as approved by the Cornell University Institutional Review Board for Human Participants. PMN isolation was performed following a protocol described previously.^13^ Briefly, PMNs were isolated by centrifugation at 500 g for 50 min using 1-Step Polymorphs (Accurate Chemical and Scientific Corporation, Westbury, NY, USA). The PMN layer was extracted and washed twice in Ca^2+^ and Mg^2+^ free HBSS to remove the polymorph residue, and any remaining red blood cells were lysed hypotonically with 1:6 and 4× PBS. PMNs were then resuspended at various concentrations of HBSS containing 0.5% HSA, 2 mM Ca^2+^, and 10 mM HEPES, buffered to 7.4. Similarly, buffy coat layer isolation was performed using Ficoll density gradient and washed with HBSS.

### Cancer Cell Capture from Spiked Buffy Coat

ZR-75-1 cells were fluorescently labeled with Cell Tracker Green (Invitrogen), a live cell dye, for 30 min at 37 °C. 100,000 or 50,000 of the labeled ZR-75-1 cells were then spiked into 1 mL of normal buffy coat and perfused through protein coated microtubes in two separate experiments. Capture efficiency was calculated using the estimated total number of cells in the microtube from 20 images recorded at random locations along the length of the microtube based on a previously published calibration.^19^

### Data Acquisition and Analysis

“Rolling” cells were defined as those observed to translate in the direction of flow with an average velocity less than 50% of the calculated hydrodynamic free stream velocity. The rolling velocity was calculated by measuring the distance a rolling cell traveled over a 30 s interval. Videos of rolling cells were taken at three randomly selected locations along the microtube. For capture experiments, adherent cells were counted after perfusion with cell-free buffer for 5 min. The quantities of cells rolling and adhering were determined by recording images at 20 randomly selected locations along the microtube. All errors are reported as standard error of the mean, and statistical analyses were performed using Prism (GraphPad Software, San Diego, CA).

### Molecular Dynamics Simulations

Free dynamics simulations were performed using the YASARA (http://yasara.org) package of MD programs with the YAMBER3 self-parameterizing force field^23^ and no external force field parameters. All simulations held the temperature and pressure constant at 300 K and 1 atm, respectively, while utilizing periodic boundary conditions, the particle mesh Ewald method for electrostatic interactions,^9^ equilibrium pH (7.4), and the recommended 7.86 Å force cutoff for long-range interactions. Complexes were solvated in a water box and neutralized by adding Ca^2+^ and Cl^−^ ions to a concentration of ~ 50 mM calcium where the water boxes were defined as cubes with lengths measuring approximately 85 Å to allow for free protein rotation. The water boxes were also allowed to adjust slightly to constrain the water density to 0.997 g/L. Conformational stresses were removed *via* short steepest descent minimizations followed by simulated annealing until sufficient convergences were reached. Free dynamics simulations were then run for 20 ns time periods.

The lectin and epidermal growth factor crystal structures of P-selectin bound to PSGL-1 (1G1S^35^), E-selectin bound to sLe^x^ (1G1T^35^), and L-selectin (3CFW) were obtained from the Protein Data Bank for use as starting atomic coordinates. Predicted uMUC1 bound to P-selectin was obtained by altering the PSGL-1 amino acid sequence of the P-selectin:PSGL-1 complex (1G1S subunit A) to the uMUC 1 sequence where two amino acids were included beyond the O-glycosylated site for consistency with the PSGL-1 crystal structure. Extra amino acids were extended to include the entire 20 U uMUC1 sequence (PDTRPAPGSTAPPAHGVTSA). In separate trials, residues S9, T10, and T18 were O-glycosylated with the carbohydrate moiety where sLe^x^ was coordinately bound to the calcium of P-selectin. To obtain starting E-selectin:uMUC1 and L-selectin:uMUC1 complexes, the E-selectin (1G1T) and L-selectin (3CFW) crystal structures were aligned on the various P-selectin:uMUC1 complexes *via* the MUSTANG algorithm.^22^

## Results and Discussion

### Selectin Ligand Expression

**Figure 1:**
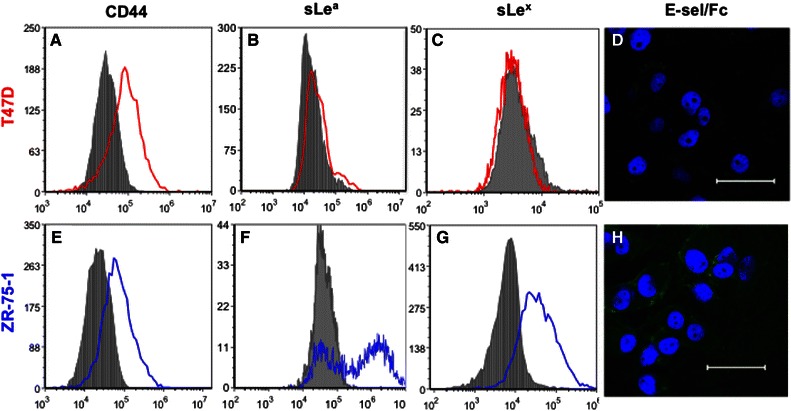
Flow cytometry histogram plots of T47D (in red) and ZR-75-1 (in blue) cells labeled with anti-CD44 mAb (a, e), anti-sLe^a^ mAb (b, f), and anti-sLe^x^ (clone CSLEX, c, g), respectively. Isotype controls are represented by solid gray peaks in each plot. (d, h) Confocal microscopy images of T47D and ZR-75-1 cells labeled with pre-conjugated recombinant human E-selectin/Fc (shown in green) and DAPI nucleic acid stain (shown in blue), respectively. Scale bar: 50 *μ*m

The surface expression of common selectin ligands and their carbohydrate binding moieties was first characterized. Both T47D and ZR-75-1 cells were found to express some amount of CD44 (Figs. 1a and 1e) while neither expressed PSGL-1 (data not shown, also suggested by Shirure *et al.* 32). These two cell lines differed in their expression of the important selectin-binding moieties sLe^a^ and sLe^x^. T47D cells exhibited very little sLe^a^ (Fig. 1b) and no sLe^x^ (Fig. 1c) while ZR-75-1 cells showed significant expression of both sLe^a^ and sLe^x^ (Figs. 1f and 1g). The differential expression of these selectin binding moieties resulted in a greater preference of ZR-75-1 over T47D cells in soluble E-selectin binding assays, as revealed by confocal microscopy (Figs. 1d and 1h). T47D cells, however, showed weak staining of bound E-selectin which may be due to the minimal surface expression of sLe^a^.

**Figure 2:**
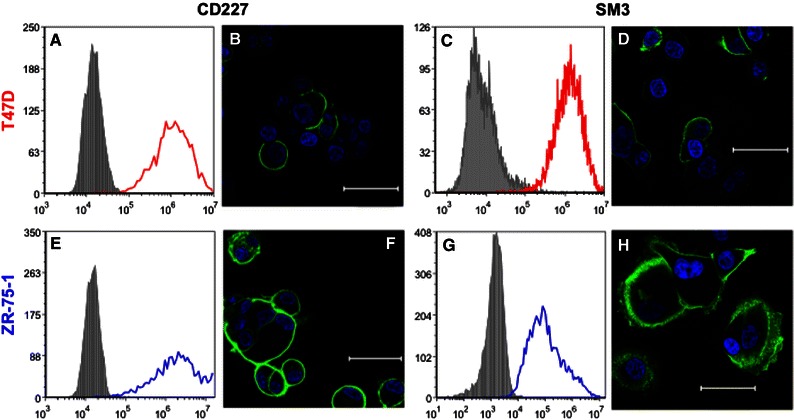
Flow cytometry histogram plots of T47D (in red) and ZR-75-1 (in blue) cells labeled with anti-MUC1 (CD227) mAb (clone HPMV, a, e) and anti-uMUC1 mAb (clone SM3, c and g). (b, f) Confocal microscopy images of T47D and ZR-75-1 cells labeled with anti-CD227 mAb (green) and DAPI nucleic acid stain (blue), respectively. (d, h) Confocal microscopy images of T47D and ZR-75-1 cells labeled with anti-uMUC1 mAb, respectively. Scale bar: 50 *μ*m

In previous research to examine the adhesive capabilities of uMUC1, we utilized the ZR-75-1 and MCF7 cells lines due to their high expression of the MUC1 glycoprotein.^14^ It was found that while both cell lines expressed MUC1, ZR-75-1 cells expressed significantly more MUC1 as indicated by SM3 antibody staining. The SM3 antibody has been shown to bind to the MUC1 core epitope corresponding to the underglycosylated form of MUC1 (uMUC1).^8^ We hypothesized that the metastatic potential of ZR-75-1 cells (highly metastatic) and MCF7 cells (weakly metastatic) correlate with the expression of uMUC1. In this study, we tested the MUC1 expression of T47D cells (weakly metastatic) compared to ZR-75-1 cells. Interestingly, both cell lines highly express MUC1 as revealed by the CD227 antibody (Figs. 2a and 2e) and exhibited high expression of uMUC1 as revealed by SM3 labeling and flow cytometry (Figs. 2c and 2g). Furthermore, both cell lines showed strong homogeneous surface staining of MUC1 (Figs. 2b and 2f) and uMUC1 (Figs. 2d and 2h) *via* confocal microscopy where ZR-75-1 cells showed greater staining for both antibodies, indicating relatively higher MUC1 and uMUC1 expression over T47D cells. While MCF7 and ZR-75-1 cells differentially expressed uMUC1, T47D and ZR-75-1 cells varied only in their expression of the carbohydrate binding moieties sLe^x^ and sLe^a^.

### Cell Rolling Assay

**Figure 3:**
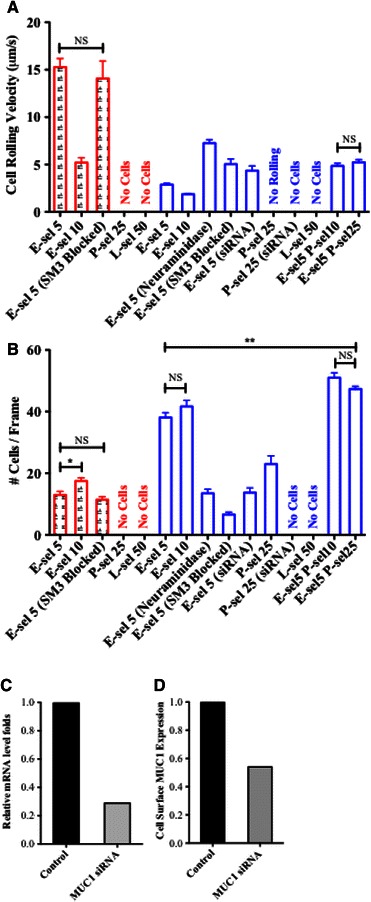
Adhesion phenotype of T47D (in red) and ZR-75-1 (in blue) cells represented by their rolling velocity on E-selectin coated surface (a) and average number of cells observed on the surface (b). E-sel, P-sel, and L-sel indicate the surface proteins E-selectin, P-selectin, and L-selectin, respectively. Numbers following the surface protein abbreviations denote the surface protein concentration in *μ*g/mL, for example “E-sel5” signifies that the surface protein E-selectin at a concentration of 5 *μ*g/mL was utilized. Combined surfaces of E- and P-selectin were utilized with constant E-selectin concentrations (5 *μ*g/mL) and varying P-selectin concentrations (10 and 25 *μ*g/mL) and are denoted as “E-sel5 P-sel10” and “E-sel5 P-sel25,” respectively. Text in parentheses following the surface protein indicators denote specific treatments of either ZR-75-1 or T47D cells where (SM3 Blocked) and (Neuraminidase) indicates that cells were incubated with either anti-SM3 neutralizing antibody or neuraminidase, respectively. (siRNA) indicates that cells were transfected with MUC1 siRNA. Absence of parentheses indicates that no such treatments were performed. Student’s *t* test was performed for all results compared to E-sel5 within each cell line. Combined surfaces E-sel5 P-sel10 and E-sel5 P-sel25 for the ZR-75-1 cell line were also paired. All significances are *p* < 0.001, unless otherwise indicated by **p < 0.01, *p < 0.05, or NS (not significant). (c) Quantification of MUC1 mRNA level knockdown efficiency *via* qPCR. (d) Quantification of cell surface MUC1 protein level knockdown efficiency *via* flow cytometry (mean fluorescence intensity index was plotted)

Lack of sLe^x^ expression generally suggests a lack of selectin adhesive interactions under flow conditions. However, T47D cells interacted with the E-selectin surface (consistent with confocal staining by soluble E-selectin and likely due to sLe^a^) which facilitated rolling events, albeit extremely fast cell rolling velocities, >15 *μ*m/s, and lower numbers of interacting cells, < 15 cells/frame, for an E-selectin concentration of 5 *μ*g/mL (Figs. 3a and 3b). Increasing the E-selectin concentration to 10 *μ*g/mL significantly decreased the cell rolling velocities to ~5 *μ*m/s and slightly increased the number of interacting cells to ~18 cells/frame indicating that cell interactions were indeed E-selectin dependent. Furthermore, perfusing cells with uMUC1 blocked with SM3 through microtubes with 5 *μ*g/mL E-selectin concentration resulted in no significant change in cell rolling velocities or number of interacting cells, suggesting that the E-selectin binding moiety responsible for these weak interactions was most likely not presented by MUC1.

Thus, the hypothesized correspondence between uMUC1 expression and metastatic potential must be amended to include the expression of sLe^x^, since T47D cells (weakly metastatic) express uMUC1 but not sLe^x^.

ZR-75-1 cells, on the other hand, showed greater interactions with the E-selectin coated microtubes yielding slower cell rolling velocities of ~3 *μ*m/s and greater numbers of interacting cells, nearly 39 cells/frame, for the 5 *μ*g/mL concentration of E-selectin (Figs. 3a and 3b), consistent with the evident expression of sLe^x^ on the cell surface. Doubling the E-selectin concentration significantly decreased the cell rolling velocities to ~2 *μ*m/s while only slightly increasing the number of interacting cells to around 40 cells/frame. SM3 blocking of ZR-75-1 cells nearly doubled the cell rolling velocity and dramatically decreased the number of interacting cells to about 6 cells/frame. This demonstrates the interruption of the E-selectin:uMUC1 interaction as first described in previous work.^14^ Inhibiting sLe^x^ moieties from the cell surface *via* neuraminidase treatment significantly increased cell rolling velocities to >7 *μ*m/s and decreased the number of interacting cells to ~ 13 cells/frame, further demonstrating the importance of the sLe^x^ binding moiety. Finally, knocking down MUC1 *via* siRNA transfection yielded an effect similar to SM3 blocking where cell rolling velocities significantly increased to 4.3 *μ*m/s and the number of interacting cells decreased to ~ 13 cells/frame. After siRNA treatment, as indicated in Figs. 3c and 3d, both mRNA and protein levels of MUC1 were reduced by at least 50% compared to the control ZR-75-1 cells.

Interestingly, perfusing ZR-75-1 cells through P-selectin coated microtubes produced around 25 interacting cells/frame (Fig. 3a), however, no cells were found rolling or firmly adhered to the surface (Fig. 3b). This interaction was abolished after siRNA knockdown of uMUC1. Therefore, the P-selectin:uMUC1 interaction facilitated tethering events where cells experienced momentary arrest to the surface followed by rapid release of the cells into the flow buffer. Furthermore, perfusing ZR-75-1 cells through combined E- and P-selectin coated microtubes resulted in around 50 interacting cells/frame, significantly more than E-selectin only surfaces (Fig. 3a), where cell rolling velocities increased nearly double compared to E-selectin-only surfaces (Fig. 3b). This further indicates the specific role of P-selectin for cell tethering rather than cell rolling. Perfusing cells through L-selectin coated microtubes did not produce any interacting cells indicating that the L-selectin:uMUC1 interaction is too weak to influence cell motion.

### Molecular Dynamic Simulations

**Figure 4:**
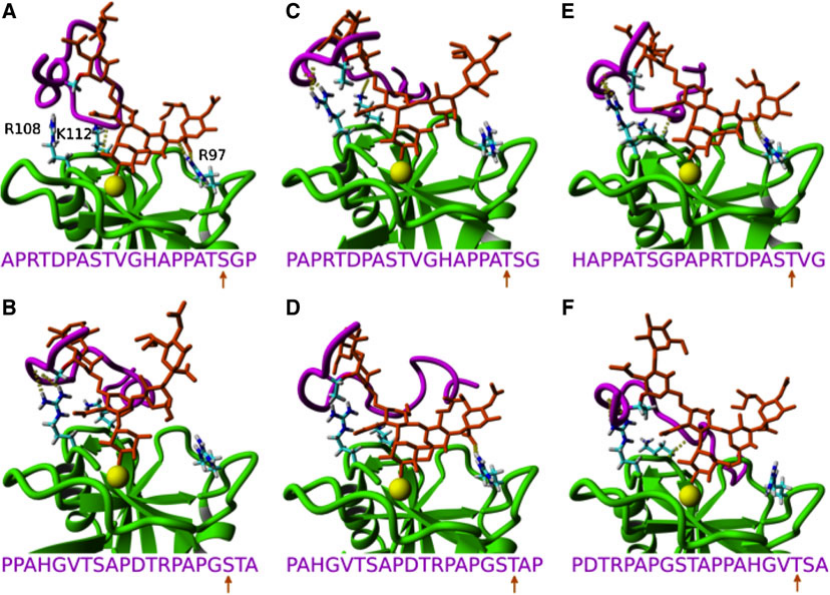
Equilibrated uMUC1 (magenta) and sLe^x^ (orange) structures bound to E-selectin (green). With respect to the uMUC1 sequence, PDTRPAPGSTAPPAHGVTSA, the top and bottom rows correspond with the “reverse” and “forward” sequence, respectively. uMUC1 residues S9 (a, b), T10 (c, d), and T18 (e, f) are glycosylated as indicated by the uMUC1 sequence and the orange arrow. The atoms of select E-selectin amino acids (R97, R108, and K112) are depicted to show varying degrees of contacting residues

To further explore the differential interactions between the uMUC1 glycoprotein and each selectin, MD simulations were used to dock several O-glycosylated variants of uMUC1 to E-, P-, and L-selectin. Figure 4 shows E-selectin:uMUC1 complexes where the S9 (Figs. 4a and 4b), T10 (Figs. 4c and 4d), and T18 (Figs. 4e and 4f) amino acid sites of the uMUC1 core epitope were O-glycosylated with a sLe^x^ moiety. Sites T3 and S19 sites were not tested because these sites should not be in a glycosylated form as suggested by SM3 binding.^8^ Each variant of uMUC1 binding indicated a different degree of residue contact. In the majority of cases, residues R108 and K112 experienced hydrogen bonding interactions, where both residues interacted with the core epitope of uMUC1 exclusively and not sLe^x^. On the other hand, residue R97 only interacted with the sLe^x^ forming hydrogen bonds in Figs. 4a, 4d, and 4e. In a single case (Fig. 4e, site T18) all three residues formed contacts and since the PSGL-1 glycoprotein is considered a P-selectin specific ligand due to enhanced interactions facilitated by the R85 and H114 residues of P-selectin,^35^ these MD results suggest that the uMUC1 glycoprotein can be regarded as an E-selectin specific ligand.

**Figure 5:**
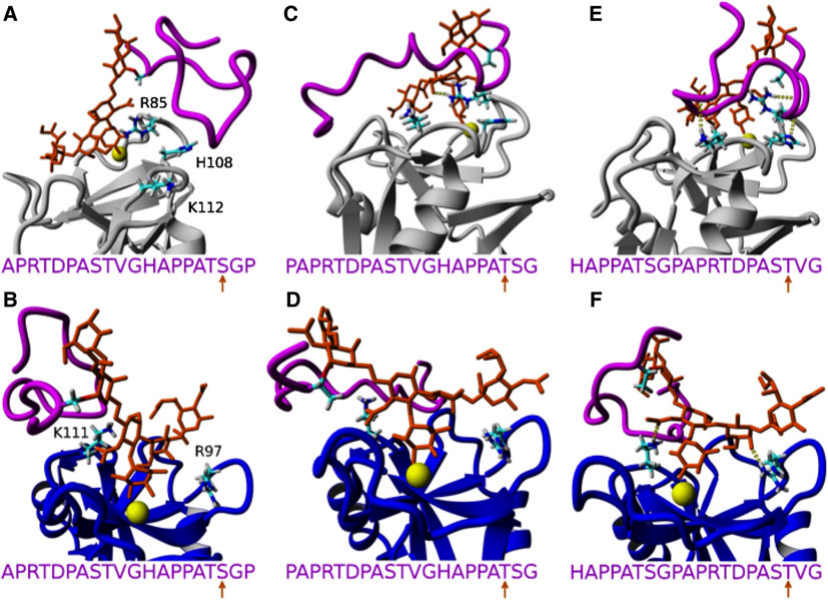
Equilibrated uMUC1 (magenta) and sLe^x^ (orange) structures bound to P-selectin (gray, top row) and L-selectin (blue, bottom row). With respect to the uMUC1 sequence, PDTRPAPGSTAPPAHGVTSA, all interactions correspond with the “reverse” sequence. uMUC1 residues S9 (a, b), T10 (c, d), and T18 (e, f) are glycosylated as indicated by the uMUC1 sequence and the orange arrow. The atoms of select P-selectin amino acids (R85, H108, and K112) and L-selectin amino acids (R97 and K111) are depicted to show varying degrees of contacting residues

Figure 5 depicts the uMUC1 interactions with P-selectin (top) and L-selectin (bottom), where one of each S9 (Figs. 5a and 5b), T10 (Figs. 5c and 5d), and T18 (Figs. 5e and 5f) glycosylated site is shown for clarity. In the case of P-selectin, residues R85, H108, and K112 formed hydrogen bonding contacts to the uMUC1 core epitope. For L-selectin, only residues R97 and K111 formed contacts where both residues contacted sLe^x^ and not the uMUC1 core epitope. Again, glycosylating the T18 site of uMUC1 yielded the most interactions for both P-selectin and L-selectin (Figs. 5e and 5f, respectively). In comparison with E-selectin, P-selectin lacked residue R97 to form contacts with sLe^x^ whereas L-selectin lacked residues R108 and K112 to form contacts with the uMUC1 core epitope. Thus, to qualitatively interpret the experimental rolling assays, E-selectin facilitates cell rolling due to its ability to contact both the uMUC1 core epitope and sLe^x^. P-selectin effectively tethers cells due to its ability to contact the uMUC1 core epitope but was unable to produce cell rolling possibly because of insufficient binding with sLe^x^. Lastly, L-selectin initiated no interacting cells due to its inability to interact specifically with uMUC1. These data suggest that uMUC1 can be considered as both an E- and P-selectin specific ligand where each selectin facilitates different roles in the metastatic adhesion cascade, whereas L-selectin most likely plays no part in functional uMUC1 binding.

### Capturing Breast Cancer Cells Using uMUC1 mAb

**Figure 6:**
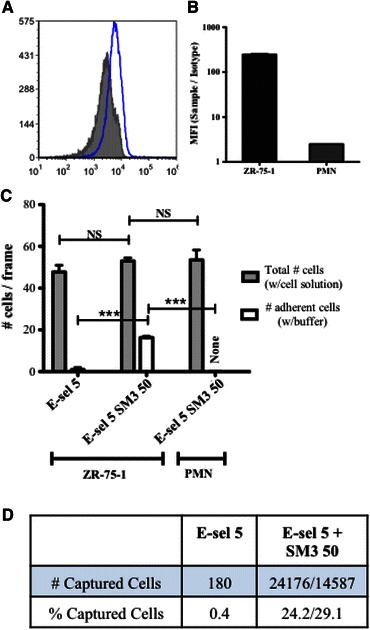
(a) Flow cytometry histogram plot of isolated PMNs labeled with anti-uMUC1 mAb (clone SM3, in blue) and isotype control (in gray). (b) Mean fluorescence intensity quantification of uMUC1 expression of ZR-75-1 cells (in black) and PMNs (in gray). (c) Average total number of ZR-75-1 cells (left and middle bar sets) and PMNs (right bar set) observed on surfaces coated with E-selectin at a concentration of 5 *μ*g/mL (E-sel5) and the combination of E-selectin and SM3 at concentrations of 5 and 50 *μ*g/mL, respectively (E-sel5 SM3 50). Columns in gray indicate the average number of cells (either ZR-75-1 or PMNs) while perfusing with cell solution and columns in white indicate the average number cells quantified after washing the surfaces with flow buffer for 5 min. Student’s *t* test was performed for both comparisons. ***p < 0.001; **p < 0.01; *p < 0.05. NS: not significant. (d) Numbers of captured ZR-75-1 cells from cancer cell spiked buffy coat on surfaces coated with E-selectin (5.0 *μ*g/mL) and the combination of E-selectin (5.0 *μ*g/mL) and SM3 (50 *μ*g/mL). Two separate experiments were performed with 100,000 cells (24,176 captured) and 50,000 cells (14,587 captured), resulting in 24.2 and 29.1% yield, respectively

Applying these insights into the selective adhesion of the uMUC1 glycoprotein, a combined E-selectin and SM3 surface was prepared to capture cancer cells. It was found that SM3 bound minimally to PMNs (Fig. 6a) *via* flow cytometry, with the mean fluorescence intensity index significantly lower compared to ZR-75-1 cells (Fig. 6b). Perfusing ZR-75-1 cells through a microtube coated with only E-selectin resulted in nearly 50 rolling cells/frame (Fig. 6c) with no adherent cells on the surface. Coating the microtube surface with both E-selectin and SM3 did not change the number of rolling cells, but allowed for firm adhesion events facilitated by SM3:uMUC1 interactions where subsequent perfusion of buffer resulted in an average of over 17 captured cells/frame. The ratio of firm adhesion events to total number of cells found on the protein coated surface was comparable to the ratio for the E-selectin/ICAM-1 combined surface as studied previously.^14^ Perfusing isolated PMNs over the same E-selectin/SM3 combined surface resulted in over 50 rolling events per frame with no adherent cells. Furthermore, since ICAM-1 also facilitates firm adhesion events with leukocytes, the E-selectin/SM3 combined surface may provide a more selective method to separate CTCs from whole blood.

Processing normal buffy coat spiked with Cell Tracker labeled ZR-75-1 cells through microtubes coated with E-selectin only or E-selectin with SM3 antibody resulted in capture efficiencies of 0.4 and 26.7%, respectively (Fig. 6d). Here, “captured” cells refers to the number of firmly adherent cells. Cells that were effectively captured by this combined surface strategy must express both the sLe^x^ glycan and the uMUC1 glycoprotein. The sLe^x^ glycan facilitates the initial tethering and rolling events due to its interactions with surface E-selectin when presented by uMUC1 or other E-selectin ligands such as CD44. Cells that initiate stable cell rolling can then form firm adhesion events due to the interaction of uMUC1 and the surface SM3. Therefore, uMUC1 and sLe^x^ expression is essential for cell capture, whether or not the sLe^x^ is found on uMUC1. However, our current and previous results indicated that sLe^x^ terminated uMUC1 is an E-selectin specific ligand that effectively contributes to cell rolling. Moreover, of the three cell lines studied here and previously, only the highly metastatic ZR-75-1 cells expressed both sLe^x^ and uMUC1 where uMUC1 was found to be decorated with sLe^x^. The capture percentage of non-cancer cells was not presented in this study due to high variance of blood cell count between donor buffy coats. Furthermore, due to the high degree of ZR-75-1 cell aggregation, the spiking cell concentration performed in this study was much higher than clinical values as well as concentrations used in our previous studies using clinically-motivated devices.^18^,^19^ Although at the proof-of-concept stage, the combined E-selectin/SM3 surface described in this study shows potential for selectively capturing CTCs expressing uMUC1.

## Conclusions

In previous work, we examined two cell lines (MCF7 and ZR-75-1) that exhibited variants of the MUC1 glycoprotein where both cell lines expressed MUC1, however only ZR-75-1 expressed the underglycosylated form uMUC1. We had previously found that uMUC1 has the ability to interact with E-selectin and ICAM-1 and, for these two cell lines, the metastatic potential corresponded with uMUC1 expression since ZR-75-1 cells are highly metastatic and MCF7 cells are weakly metastatic.^14^ Here, we further explored the function of uMUC1 during the metastatic adhesion cascade by extending the study to the T47D cell line (weakly metastatic). It was found by examining various E-selectin ligands that T47D and ZR-75-1 cells differed only in their expression of the E-selectin binding moiety sLe^x^ where T47D cells lacked this expression. Without sufficient sLe^x^, T47D cells showed little interaction with E-selectin coated microtubes, resulting in extremely fast rolling velocities and low numbers of interacting cells. Thus, for these three cell lines, the metastatic potential seems to correlate with the expression of uMUC1 as well as sLe^x^.

ZR-75-1 cell interactions with E-selectin, however, were highly dependent on uMUC1 expression where cell velocities significantly decreased when uMUC1 was blocked with either the SM3 antibody or uMUC1 siRNA knockdown. Moreover, uMUC1 interactions with P-selectin exclusively facilitated cell tethering events on P-selectin-only and E- and P-selectin combined surfaces whereas L-selectin surfaces produced no cell interactions. Utilizing MD simulations, E-selectin was shown to interact with uMUC1 *via* three distinct residue contacts to both the uMUC1 core epitope and sLe^x^. On the other hand, P-selectin possessed three distinct contacts that interacted with only the uMUC1 core epitope and L-selectin only interacted with sLe^x^. These MD data suggest that specific interactions with both the core epitope and sLe^x^ is required to produce cell rolling events whereas interactions with just the core epitope produce tethering events and interactions with only the carbohydrate unit do not produce any cell interactions.

Based on these observations, we propose a combined surface of E-selectin and SM3 to preferentially capture cells exhibiting uMUC1. It was found that the E-selectin/SM3 microtubes captured approximately 27% of the number of interacting cancer cells. Since PMNs are not targeted by the SM3 mAb, it is expected that this E-selectin/SM3 surface strategy may offer a novel and viable method to selectively isolate cancer cells from whole blood.
